# Proton Therapy for Mandibula Plate Phantom

**DOI:** 10.3390/healthcare9020167

**Published:** 2021-02-04

**Authors:** Güler Burcu Senirkentli, Fatih Ekinci, Erkan Bostanci, Mehmet Serdar Güzel, Özlem Dağli, Ahmad M. Karim, Alok Mishra

**Affiliations:** 1 Department of Pediatric Dentistry, Baskent University, Ankara 06810, Turkey; gbsenirkentli@baskent.edu.tr; 2 Department of Physics, Gazi University, Ankara 06500, Turkey; fatih.ekinci2@gazi.edu.tr; 3 Computer Engineering Department, Ankara University, Ankara 06830, Turkey; ebostanci@ankara.edu.tr (E.B.); mguzel@ankara.edu.tr (M.S.G.); 4 Department of Neurosurgery Gamma Knife Unit, Gazi University, Ankara 06850, Turkey; ozlemdagli@gazi.edu.tr; 5 Computer Engineering Department, Ankara Yıldırım Beyazıt University, Ankara 06830, Turkey; ahmet.sarikahya@gmail.com; 6 Faculty of Logistics, Molde University College-Specialized University in Logistics, 6402 Molde, Norway; 7 Software Engineering Department, Atilim University, Ankara 06830, Turkey

**Keywords:** proton treatment, biomaterials, bragg peak, mandible plate phantom, dental tumour, paediatric dentistry

## Abstract

Purpose: In this study, the required dose rates for optimal treatment of tumoral tissues when using proton therapy in the treatment of defective tumours seen in mandibles has been calculated. We aimed to protect the surrounding soft and hard tissues from unnecessary radiation as well as to prevent complications of radiation. Bragg curves of therapeutic energized protons for two different mandible (molar and premolar) plate phantoms were computed and compared with similar calculations in the literature. The results were found to be within acceptable deviation values. Methods: In this study, mandibular tooth plate phantoms were modelled for the molar and premolar areas and then a Monte Carlo simulation was used to calculate the Bragg curve, lateral straggle/range and recoil values of protons remaining in the therapeutic energy ranges. The mass and atomic densities of all the jawbone layers were selected and the effect of layer type and thickness on the Bragg curve, lateral straggle/range and the recoil were investigated. As protons move through different layers of density, lateral straggle and increases in the range were observed. A range of energies was used for the treatment of tumours at different depths in the mandible phantom. Results: Simulations revealed that as the cortical bone thickness increased, Bragg peak position decreased between 0.47–3.3%. An increase in the number of layers results in a decrease in the Bragg peak position. Finally, as the proton energy increased, the amplitude of the second peak and its effect on Bragg peak position decreased. Conclusion: These findings should guide the selection of appropriate energy levels in the treatment of tumour structures without damaging surrounding tissues.

## 1. Introduction

The use of accelerated protons in radiotherapy was initially proposed by Robert Wilson in 1946 [1]. Lawrence et al. conducted the first research into the use of protons for treatment in 1954 [2]. The first patient-based studies of protons began in 1990 at the University of Loma Linda [3]. Since the proton is a heavily charged particle, it loses its energy slowly but continuously by scattering at small angles as it travels through the matter. The Bragg peak occurs where protons have the highest rate of energy loss. Due to the tumour volume, it is not sufficient to send the proton beam with only one energy level to the target. Therefore, proton beams of different energies must be sent to the target [4]. To determine the energy range, calculations were made using different phantoms using simulation software [5,6,7,8,9,10,11].

Routine cancer treatment uses only one or a combination of three treatment modalities: surgical treatment, chemical treatment (chemotherapy) and radiation treatment. The majority of cancer patients receive radiation in all or part of their treatment [12]. Radiation therapy is a treatment method developed by using ionizing radiation to destroy malignant tumour cells or to prevent the growth or proliferation of these cells [13].

Proton therapy is a method of radiation therapy distinguished by X-ray modalities by the Bragg peak. With this technique, radiation is administered to the target, while ensuring that normal tissues are not affected by radiation [14]. This has been hypothesized to improve the therapeutic ratio of treatment in several disease sites [15].

The anatomical complexity of the head and neck presents a challenging treatment pattern due to the proximity of tumours to the surrounding normal tissues [16]. Jawbones, the maxilla and mandible and related tissues can be the site of a multitude of neoplastic conditions. These tumours have a predilection for the entire facial region; however, jaw and odontogenic tumours tend to affect the mandible more than the maxilla [17]. Malignant tumours of the mandible and maxilla are sorted primary tumours that originate within the mandible and secondary lesions, mainly oral cancers and metastatic lesions, that involve the mandible secondarily [18]. Despite recent advances in surgical techniques, radiotherapy and chemotherapy, the treatment of locally advanced head and neck cancers in the oral cavity have been challenging in oncology [19].

Oral cancers spread to local and deeper tissues through neuronal, lymphatic pathways and anatomical spaces [20]. Tumours originating from the mandible around the tooth first shows spread to other oral tissues such as the upper surface of the mandible and secondary tumours to the neck, periodontal membrane, the attached gingiva and to the other soft tissues in the oral cavity. In addition to the local extension, it may invade deeper tissues [21]. Since odontogenic tumours in the mandible are frequently involved in the posterior region of the mandible, and considering the complexity of the anatomy of the region [22], this region is focused in this study.

When the topographical structure of the mandibular molar region is considered, the layers can be listed as skin, parotid, SMAS, masseter, buccal adipose tissue, buccal mucosa, gums, cortical bone and cancellous bone. Considering this complex anatomy, the possibility of radiotherapy being applied outside the mouth to affect all these tissues and the subsequent complications should be taken into consideration. Head and neck radiotherapy is associated with significant acute and late toxicities, including taste disorders, mucositis, dysphagia, weight loss and xerostomia [23].

Radical surgery, chemotherapy, radiotherapy or combinations of these treatments are used in the treatment of oral cancers [24]. One of the adverse effects of radiotherapy for head and neck cancer is radiation-induced osteomyelitis or osteoradionecrosis of the jawbone. Several clinical and physical factors have been reported to be associated with the risk of osteoradionecrosis, which may include patient, tumour and treatment-related factors. The overall effect of radiation therapy on oral tissues and craniofacial skeletal growth, a spectrum of minor to major complications, should be considered for all paediatric and adult patients undergoing such treatment [25,26,27]. Oral complications of radiotherapy are referred to as oral mucositis, dysgeusia, infectious diseases and xerostomia associated with loss of glandular function. The incidence of these complications increases with the dose of radiotherapy [28].

Improvements in radiation dose distribution, particularly the development of Intensity Modulated Radiation Therapy (IMRT), have increased the therapeutic rate of treatment by reducing the incidence of toxicity by selective separation of certain organs at risk [29]. Numerous studies show that severe treatment toxicities have been associated with increased doses to sites such as the base of the mouth, submandibular glands, parotid glands, oral cavity and other sites. Optimized dose distribution reduces clinical toxicity [30].

In this study, we calculate the required dose rates for optimal treatment of tumoral tissues using proton therapy in the treatment of defective tumours seen in the mandible such as squamous cell carcinoma to protect the surrounding soft and hard tissues from unnecessary radiation and also prevent complications of radiation. Bragg curves of therapeutic energized protons for two different mandible (molar and premolar) plate phantoms were calculated and compared with similar calculations in the literature. The results were found to be within acceptable deviation values. Finally, Bragg curves and peak positions, lateral straggle/range and recoil curves were obtained for both mandibular zone plate phantoms. Based on the results achieved, we tried to determine the proton energy to be selected according to the tumour position. Lateral straggle and range values in the tissue layers of the mandibular region were determined. The mandible plate phantom results were compared.

The rest of the paper is structured as follows: Section 2 elaborates the details of the models used and the approach used to compute Bragg curves, followed by Section 3 where the results are presented. A discussion is given in Section 4 on the findings of our study and finally, the paper is concluded in Section 5.

## 2. Material and Method

Organs at risk with specified dose constraints were contoured to calculate the appropriate radiation dose, including the cheek, masseter muscle, parotid gland, oral mucosa, gingiva, mandibular cortical and cancellous bone, SMAS and saliva and air. The thickness and density calculations of all contours were made based on previous studies in the literature [31]. Tissue thicknesses for the molar and premolar tooth region were calculated for this study and two different optimal dosages were obtained for these regions.

The Linear Energy Transfer (LET) calculations of the protons in the target material were performed with the help of Monte Carlo based TRIM (Transport of Ions in Matter) and SRIM (Stopping and Range of Ions in Matter) simulation software. TRIM is a software that computes the stopping power and range of ions within the target using quantum mechanical methods for ion-atom collisions. TRIM takes all kinetic events related to the energy loss processes of ions, particularly ionization [32], into account for the computations performed.

**Figure 1:**
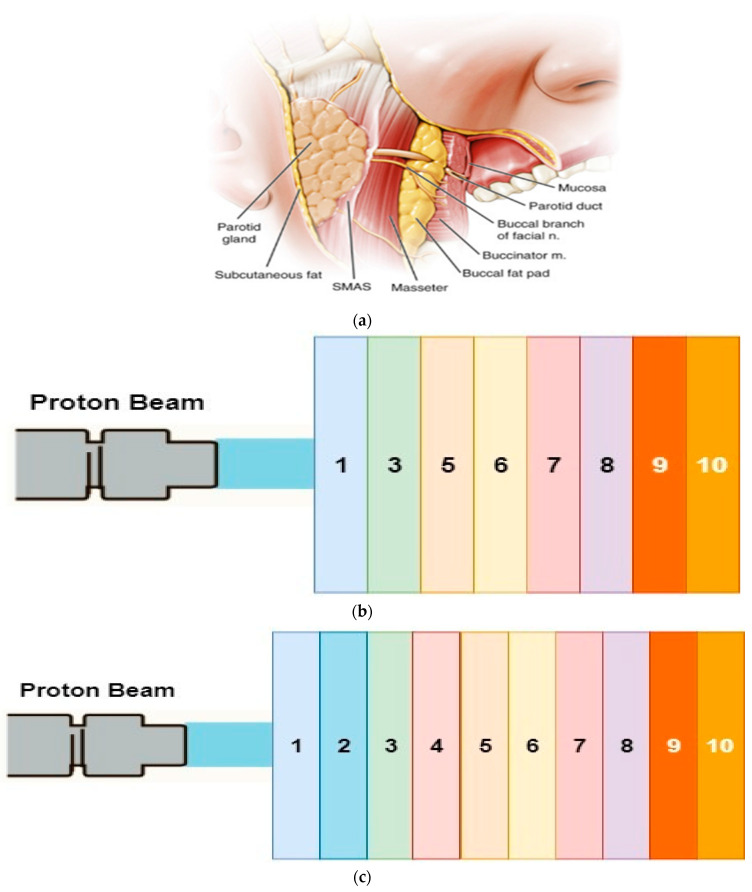
(**a**) A cross-sectional view of the focused mouth area (**b**) Mandibular region tooth plate premolar phantom section (**c**) Molar phantom section of mandibular region tooth plate. Layers (1–10) are explained in detail in Table 1.

In the calculations, the proton beam (105 protons) was sent to the target with statistical deviations within acceptable limits. As shown in Figure 1, the calculations were carried out considering two situations. The first is the mandibular plate section containing the thickness, mass and atomic density of the anatomical layers, taking into account the physiology of the mandible, the mandibular premolar region and the second molar region.

All layers of the jawbone were taken into account using the thicknesses of the SRIM database. The atomic percentages, atomic densities and densities of all biological layers forming the jawbone were obtained from the SRIM compounds database and are presented in Table 1.

## 3. Results

Bragg curves normalized to the maximum dose in the water phantom of 80, 100, 120, 140, 160, 180, 200 MeV energies were compared with the literature to test the accuracy of the calculations to find suitable doses for proton therapy in the mandible [33,34]. In comparison, it is clear that the average 2.7% difference is generally not significant and is within acceptable limits (<5%) in the medical field. Deviations above the acceptable difference are within acceptable limits in the literature, considering inhomogeneity effects and Monte Carlo-based probabilities. Our calculations were found to be consistent with the studies in the literature.

### 3.1. Bragg Peaks

Bragg curves were obtained by excluding the parotid gland and masseter layers in the mandibular premolar plate phantom section (Figure 1b). The energy of proton beams with 41–45 MeV energy was increased by 0.1 MeV and the changes in all layers of tooth plate phantom were investigated.

**Figure 2:**
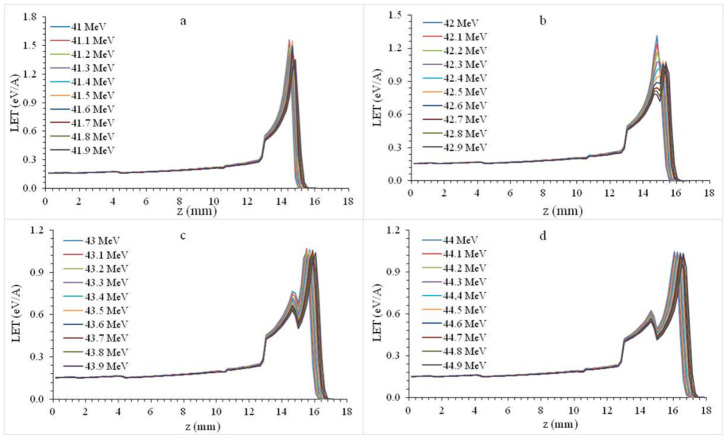
Bragg curves formed by 41–44.9 MeV (**a**–**d**) energy protons in premolar plate phantom.

In Figure 2a, the 41–41.9 MeV proton beam formed a Bragg peak in the cortical bone. An increase in the Bragg Peak amplitude (LET for the beam) can be observed in the transition from the gingival to the higher density structures. In Figure 2b, 42–42.3 energy protons form a Bragg peak in the cortical bone, while 42.4–42.9 energy protons form a Bragg peak in the cancellous bone. A decrease in LET was observed in the transition from a denser medium such as cortical bone to a less dense medium such as cancellous bone. In Figure 2c,d, 43–44.9 MeV protons formed the Bragg peak in the cancellous bone. The secondary LET increase in the cortical bone region was the highest peak increase point after the Bragg peak; we call this marginal increase the second peak.

**Figure 3:**
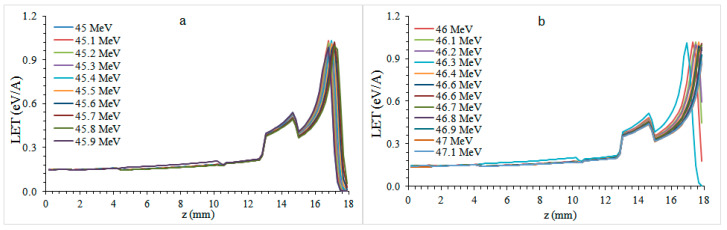
Bragg curves formed by 45–47.1 MeV (**a**,**b**) energy protons in premolar plate phantom.

In Figure 3a, the 45.9 MeV energy proton beam continued to transmit LET into the oral cavity approximately 0.2 mm after the Bragg peak.

In Figure 3b, proton beams with 46–47.1 MeV energy leave their post-peak energy into the oral cavity. As the energy of the proton beam decreased from 41 MeV to 47.1 MeV, the Bragg peak amplitude decreased from 1.5577 eV/A to 0.8787 eV/A, which was 44%.

In the second calculations using mandibular tooth plate molar phantom (Figure 1c), a total of 40 different energy proton beams were used in the 89.8–93.7 MeV energy range. With this wide energy range, the maximum LET was transferred over an area of 60.99–64.9 mm.

**Figure 4:**
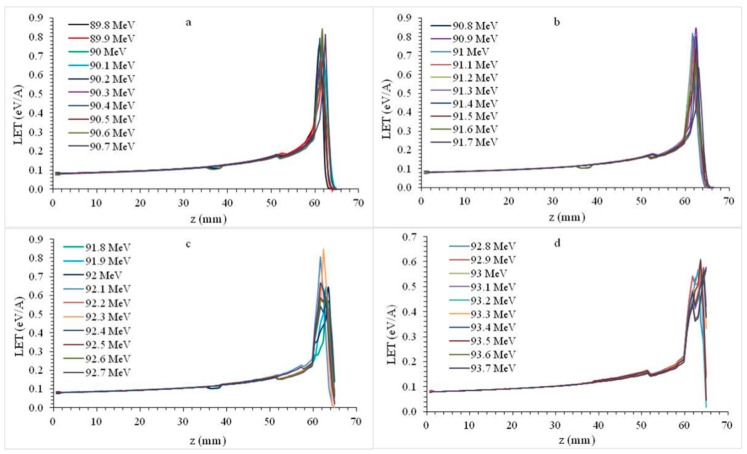
Bragg curves formed by 89.8–93.7 MeV (**a**–**d**) energy protons in molar plate phantom.

Referring to Figure 4a, maximum LET was transferred by forming a Bragg peak at the last 0.87 mm of the cortical bone and 0.65 mm of the cancellous bone. As seen in Figure 4b–d, Bragg peaks formed along the cancellous bone were formed. Referring to Figure 4d, the LET transfer after the Bragg peak occurred in the oral cavity. Bragg curve profile fractures were observed due to LET decrease in the cancellous bone region, which was less dense than cortical bone. As the energy of the proton beam decreased from 89.8 MeV to 93.7 MeV, the Bragg peak amplitude decreased from 0.7567 eV/A to 0.5453 eV/A, which was 28%.

### 3.2. Lateral Range and Straggle

**Figure 5:**
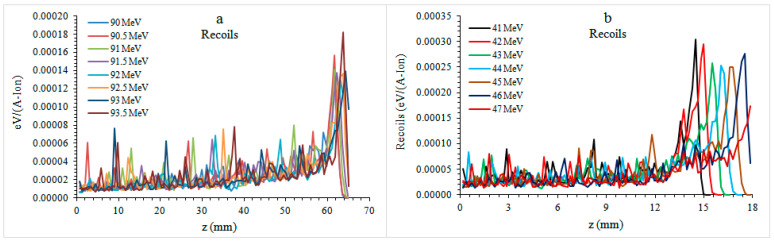
Recoils curves of different energy proton beams in tooth plate molar (**a**) and premolar (**b**) phantoms.

According to the results presented in Figure 5, the proton beam collided in the atomic dimension as it progressed through the layered structure of varying density. Most of the atomic collisions occurred at the recoils peak, which is at the same position with the Bragg peak. The mandible plate fluctuated further as the collision energy increased at the low atomic dimension at the phantom inlet. Molar plate phantom recoils (3.04924–1.39057) × 10^−4^ eV/(A-ion) occurred as the energy was reduced by 54%. In the premolar plate phantom, recoils (1.40564–0.18248) were observed as × 10^−4^ eV/(A-ion) and decreased by 87% as the energy increased.

Ionization was a thousand times larger than atomic collisions. The lateral range was 0.9 to 0.4 mm and lateral straggle was between 1.5 and 0.8 mm.

**Figure 6:**
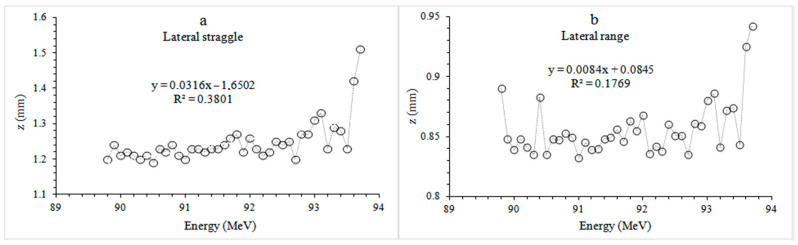
Recoils curves of different energy proton beams in tooth plate molar phantoms. (**a**) Lateral straggle (**b**) Lateral range. Circles indicate lateral straggle and range for different energy levels.

When the lateral straggle and range values of the mandible bone molar plate phantom were examined (Figure 6), it was seen that the proton beam was spread by approximately 1.20 mm at 89.8 MeV. The beam spread to approximately one-third of the cortical bone with an average thickness of 1.92 mm. The 93.7 MeV proton beam resulted in 1.51 mm lateral straggle inside the 3.11 mm thick cancellous bone, a spread to about the half of the tissue. In the lateral range, the protons with energy of 89.8 MeV were 0.89 mm and the protons with energy of 93.7 MeV were 0.94 mm, from the phantom entrance to the point where all energy was released.

**Figure 7:**
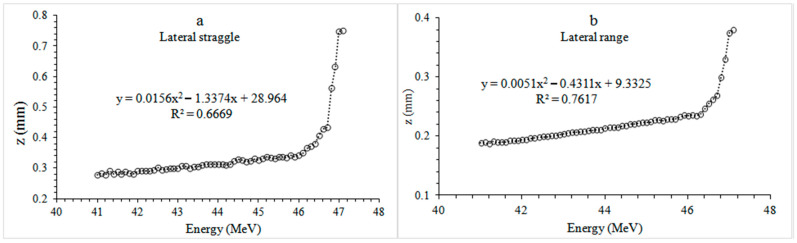
Recoils curves of different energy proton beams in dental plate phantoms. (**a**) Lateral straggle (**b**) Lateral range. Circles indicate lateral straggle and range for different energy levels.

When the lateral straggle and range values of the premolar plate phantom (Figure 7) were examined, it was seen that the proton beam spread by 0.28 mm at 41 MeV, which is a spread to approximately one-sixth of the cortical bone with an average thickness of 1.92 mm. A 47.1 MeV proton beam was spread 0.75 mm laterally and spread to about a quarter of the 3.11 mm thick cancellous bone. When looking at the lateral range, the proton of 41 MeV was 0.38 mm, while the proton of 47.1 MeV was 0.38 mm, from the entrance to the phantom to the point where it released all its energy.

## 4. Discussion

Surgery is one of the first treatment modalities for most oral cancers [33]. In the past, mandibular resection as part of the excision of many oral cancers was the standard method. It was initially thought to be necessary for adequate tumour clearance [34]. The mandible is important for dentition and chewing as well as speaking, swallowing and maintaining an effective airway. With advances in imaging and reconstruction techniques, applications that are more sophisticated can be employed [35].

Proton beam therapy is one of the most advanced types of radiotherapy [36]. The physical and radiobiological properties of protons allow a superior dose distribution as compared to current photon (X-ray) radiotherapy, thereby minimizing the dose to normal tissues and significantly reducing acute and late side effects [37]. Conventional radiation therapy faces challenges from side effects due to a relatively high entrance dose as well as a non-zero exit dose. By contrast, proton therapy has a substantially lower entrance dose and no exit dose, reducing the damage to healthy tissues surrounding a tumour [38]. Since the use of proton beam radiotherapy, the number of diseases which can be curatively treated has increased [39].

Dosimetry studies have shown an advantage for proton beam therapy compared with photon-based therapy for the treatment of oropharyngeal carcinoma in terms of sparing of multiple critical organs, especially with the use of smaller proton spot sizes [39,40]. In our study, bone and soft tissue thicknesses were established using previous studies, but a more standardized method can be developed by using advanced imaging methods to calculate the appropriate radiation dose. Reduction in toxicity potential after radiotherapy using proton therapy was reported in studies [41,42]. In some studies, a significant reduction of the risk of side effects was observed in nearly 70% of the cases, according to existing and validated normal tissue complication probability models [43]. The method mentioned in this study is designed in phantom jaw model. In future studies, treatment models in which radiation effects are further optimized can be applied by working with a patient group and calculating personal tissue thicknesses.

The weakness of this study is that the method mentioned here is designed for the jaw phantom model. As mentioned above, treatment models in which radiation effects are optimized can be applied by working with a patient group and calculating personal tissue thicknesses.

In head and neck cancer, in particular, proton beam therapy is uniquely suited for the complex anatomy of tumours and sensitive surrounding organs [44,45]. A previous study reported acute toxicities and early outcomes following the use of proton beam therapy for paediatric head and neck malignancies. As a result of the study, it has been found that there are low acute toxicity rates with proton beam therapy, and it is safe for the paediatric patient population in accordance with previous studies [46].

## 5. Conclusions

The unique traits of proton therapy allow for greater normal tissue sparing without sacrificing target coverage when irradiating the head and neck for both paediatric and adult patients. This dosimetric advantage seemingly translates into lower rates of acute treatment-related toxicity, in addition to reducing the radiation doses applied to proton therapy.

In the presented study, Bragg, lateral straggle/range and recoil curves of therapeutic energized protons were calculated by Monte Carlo simulation by considering two different tooth plate section phantoms, namely premolar and molar. The calculations were made considering the tooth layers and assumed that the dental tumour was formed in the cancellous bone. The Bragg peak, which is the maximum LET transfer point for both tooth plate phantoms, was formed along the average length of 3.11 mm of the cancellous bone. Thus, maximum LET transfer throughout the tumour volume was achieved.

The proton beam has transferred 51% more LET to premolar phantom than the molar phantom at initial energy levels. Similarly, for higher energy levels, 34% more LET was transferred to the premolar phantom than the molar phantom. The main reason behind this is that, as the range increases more LET transfer occurred along with the increase in LET as the beam passes through the layers. Ionizations increased due to the density variations in the target layers. The densest structure in the phantom that we used is the cortical bone. In this layer, the proton beam transferred a large amount of LET, and we called this peak, occurring due to large LET transfer, the second peak. The second peak is the second highest LET transfer location in the Bragg curve. This second peak behaves like the Bragg peak since peak amplitude decreases as the energy of the proton beam increases. One can observe an increase in the amplitude of the second peak as the thickness of the cortical bone increases. This means that the thickness of the cortical bone has reduced the range of the proton beam. Since the thickness and the number of layers is less in the premolar phantom, the energy of the proton beam was 52% less. The proton beam created 32.4% and 49.5% more lateral range in the initial and higher energy levels, respectively. Likewise, we observed 25.8% and 37.6% more lateral straggle for initial and higher energy levels in the molar phantom.

Proton therapy can be considered an alternative to invasive surgical treatment in the treatment of tumours in the jaw bones. We believe that our findings will be employed as a guide to determining the dose levels to be used in the treatment.

Main points: 1. In this study, we calculated the required dose rates for optimal treatment of tumoural tissues using proton therapy in the treatment of defective tumours seen in the mandible. 2. Mandibular tooth plate phantoms were modelled for the molar and premolar areas and Monte Carlo simulation was used to calculate the Bragg curve, lateral straggle/range and recoil values of protons remaining in the therapeutic energy ranges. 3. These findings should guide the selection of appropriate energy levels in the treatment of tumour structures without damaging surrounding tissues.

## Figures and Tables

**Table 1:** Atomic percentage, atomic density and density of the biological layers forming from the mandible [32].

Biomaterial	Chemical Composition (%)	Atomic Density (10^22^ atom/cm^3^)	Density (g/cm^3^)
1. Skin	H 10; O 59.4; C 25; N 4.6; S 0.3; Cl 0.3; P 10.3; Na 0.2; K 0.1	9.88	1.02
2. Parotid gland	H 62.5; C 16.4; N 1.27; O19.6; S 0.037; Cl 0.016; Na 0.025; P 0.019	10.32	1.02
3. SMAS	H 58.3; C 37.4; N 1.45; O 1.89; F 0.532; Ca 0.266	10.65	1.027
4. Masseter muscle	H 52.6; C 8.9; N 1.6; O 26.6; S 5.85; Cl 1.76; K 0.64; P 0.404	10.11	1.05
5. Buccal Fat	H 63.4; C 28.4; N 0.304; O 7.77; Cl 0.018; Na 0.011	10.35	0.92
6. Mucosa	H 10.1; C 77.5; N 3.50; O 5.23; F 1.74;Ca 1.83	5.24	1.028
7. Saliva	H 66.6; O 33.3	10.02	1
8. Gum	H 52.6; C 32.9; N 0.862; O 7.89; Cl 1.72; Mg 3.63	8.88	1
9. Cortical bone	H 39.2; C 15; N 3.48; O 31.6; S 0.108; P 3.86; Ca 6.53; Mg 9.57	9.94	1.92
10. Cancellous bone	H 57.7; C 23; N 1.36; O 15.7; S 4.27; P 0.752; Ca 1.26; Fe 1.23	10.42	1.18

## Data Availability

Not applicable.
